# Red Blood Cell Passage of Small Capillaries Is Associated with Transient Ca^2+^-mediated Adaptations

**DOI:** 10.3389/fphys.2017.00979

**Published:** 2017-12-05

**Authors:** Jens G. Danielczok, Emmanuel Terriac, Laura Hertz, Polina Petkova-Kirova, Franziska Lautenschläger, Matthias W. Laschke, Lars Kaestner

**Affiliations:** ^1^Institute for Molecular Cell Biology, Saarland University, Homburg, Germany; ^2^Experimental Physics, Saarland University, Saarbrücken, Germany; ^3^Leibniz Institute for New Materials, Saarbrücken, Germany; ^4^Institute for Clinical and Experimental Surgery, Saarland University, Homburg, Germany; ^5^Theoretical Medicine and Biosciences, Saarland University, Homburg, Germany

**Keywords:** RBC deformation, Piezo1, hSK4 (KCNN4), Ca^2+^ imaging, microfluidics, dorsal skinfold chamber

## Abstract

When red blood cells (RBCs) pass constrictions or small capillaries they need to pass apertures falling well below their own cross section size. We used different means of mechanical stimulations (hypoosmotic swelling, local mechanical stimulation, passing through microfluidic constrictions) to observe cellular responses of human RBCs in terms of intracellular Ca^2+^-signaling by confocal microscopy of Fluo-4 loaded RBCs. We were able to confirm our *in vitro* results in a mouse dorsal skinfold chamber model showing a transiently increased intracellular Ca^2+^ when RBCs were passing through small capillaries *in vivo*. Furthermore, we performed the above-mentioned *in vitro* experiments as well as measurements of RBCs filterability under various pharmacological manipulations (GsMTx-4, TRAM-34) to explore the molecular mechanism of the Ca^2+^-signaling. Based on these experiments we conclude that mechanical stimulation of RBCs activates mechano-sensitive channels most likely Piezo1. This channel activity allows Ca^2+^ to enter the cell, leading to a transient activation of the Gardos-channel associated with K^+^, Cl^−^, and water loss, i.e., with a transient volume adaptation facilitating the passage of the RBCs through the constriction.

## Introduction

The physiological function of the Gardos-channel, a Ca^2+^-activated K^+^-channel (Gardos, 1958) in the red blood cell (RBC), that was later identified as the hSK4 (KCNN4) channel (Hoffman et al., 2003), was obscure for decades. It was regarded as a RBC suicidal mechanism (Andrews and Low, 1999; Kaestner and Bernhardt, 2002; Lang and Qadri, 2012) including the process of dehydration associated with Gardos-channel activity (Begenisich et al., 2004; Lew et al., 2005). Such a concept was mainly consequent to the observation that an increase in the intracellular RBC Ca^2+^-concentration was associated with numerous processes leading in their synergistic effects to cell death (Bogdanova et al., 2013). The Gardos-channel fits well into this concept as it requires Ca^2+^ to be activated and its activation results in K^+^-loss associated with loss in Cl^−^ and water and hence in cell shrinkage. In addition within the recent years hereditary anemic disorders have been associated with mutations in the Gardos-channel (Glogowska et al., 2015; Fermo et al., 2017).

Furthermore, several sources of Ca^2+^-entry have been identified in RBCs, like a non-selective voltage-activated cation channel (Kaestner et al., 2000), Ca_V_2.1 (Andrews et al., 2002), TRPC6 (Foller et al., 2008), VDAC (Bouyer et al., 2011), or NMDA-receptors (Makhro et al., 2013). Complementary, an increased activity of these channels has been associated with pathophysiological conditions such as prostaglandin E_2_ and lysophosphatidic acid release from activated platelets (Li et al., 1996; Yang et al., 2000; Kaestner et al., 2004, 2012), malaria infection (Bouyer et al., 2011) and anemias like sickle cell disease or rare anemia (Hänggi et al., 2014; Hertz et al., 2017).

During the past years a new player was discovered, Piezo1, a mechano-sensitive non-selective cation channel (Coste et al., 2010; Gottlieb and Sachs, 2012; Gottlieb et al., 2012) that is also abundant in RBCs (Zarychanski et al., 2012; Albuisson et al., 2013; Kaestner, 2015). This discovery initiated studies to link mechanical stress associated Piezo1 activity with RBC volume regulation by Gardos-channel activity (Gallagher, 2013; Faucherre et al., 2014; Cahalan et al., 2015). However, these studies have either been performed in animal models (Faucherre et al., 2014; Cahalan et al., 2015) or in relation to pathophysiological conditions (Glogowska and Gallagher, 2015). Here, we investigate healthy human RBCs subjected to different forms of mechanical stimulation using intracellular Ca^2+^ changes as a read-out parameter and compare their behavior when Piezo1 is inhibited by the toxin GsMTx-4 (Bae et al., 2011).

## Materials and methods

### Human RBCs

Blood sampling from humans was approved by the ethical committee (Ärztekammer des Saarlandes, approval number 132/08) upon informed consent. Blood was collected in 9 mL heparin tubes (Vacuette, Becton, Dickinson and Co., Franklin Lakes, NJ, USA) and immediately used for measurements as recommended (Makhro et al., 2016). All treatments and measurements of RBCs were performed in a laboratory of biological safety level S2. Blood was washed three times in Tyrode solution containing in mM: 135 NaCl, 5.4 KCl, 10 Glucose, 10 HEPES, 1.8 CaCl_2_, 1 MgCl_2_, pH 7.35 adjusted with NaOH, 300 mOsmol/kg H_2_O. The washing procedure was based on 3 min centrifugation with 1,000 × g, supernatant and buffy coat were removed by aspiration. All experiments were performed at least three times with RBCs from three different donors.

### Cell staining and pharmacological interventions

During the staining procedure and the experiments, RBCs were kept in Tyrode solution and incubated with Fluo-4, AM or Calcein Red-Orange, AM (both ThermoFisher Scientific, Waltham, MA, USA) at a concentration of 5 μM (from a 1 mM stock solution in dimethyl sulfoxide (DMSO) containing 20% Pluronic F-127) for 1 h. After the staining, cells were washed three times by 3 min centrifugation with 1,000 × g. Then RBCs were plated on coverslips and 20 min were allowed for sedimentation and deesterification of the Fluo-4. Mechano-sensitive channels and the Gardos-channel were inhibited by GsMTx-4 and TRAM-34, respectively. GsMTx-4 was purchased from Alomone Labs (Jerusalem, Israel) and TRAM-34 from Sigma-Aldrich (St. Louis, MO, USA). For both substances, stock solutions were prepared at 1 mM in aqua dest. Further applications were performed at concentrations indicated in the experimental description.

### Confocal imaging

Confocal imaging was performed with a 2D-array kilobeam scanner (Infinity-4, VisiTech Int., Sunderland, UK) as previously described (Danielczok et al., 2017). In short, excitation was performed with a 491 nm DPSS laser (Calypso, Cobolt, Solna, Sweden). The confocal scanner was attached to an inverted microscope (TE2000-U, Nikon, Tokyo, Japan) utilizing a 60x objective (NA 1.4) and using a confocal aperture of 64 μm. Image acquisition was done with an EM-CCD camera (iXon887, Andor, Belfast, UK) cooled down to −50°C and used in frame transfer mode. Exposure time was 2,000 ms for the measurements at stasis and 500 ms for the microfluidic experiments at a 1 × 1 binning and a pixel read-out of 10 MHz. A pre-amplification of 2.4 and an EM-gain of 180 was applied. The entire measurement process was software controlled (VoxCellScan, VisiTech Int., Sunderland, UK). The data analysis concept was previously described (Wang et al., 2013) and image analysis is detailed below. Further data analysis and determination of the maximal cellular response was processed in Igor Pro 6.2 (WaveMetrics, Portland, Oregon, USA) with custom made macros.

### Mechanical stimulation

Mechanical stimulation was done either by application of a hypoosmotic solution or by touching the RBC with a micropipette. For the hypoosmotic solution Tyrode solution (see section Human RBCs above) was diluted by aqua dest. until an osmolarity of 200 mosml was reached. Osmolarity was measured using a vapor pressure osmometer (Vapro, Wescor, South Logan, UT, USA). Solution was applied by a local gravity driven perfusion system where the continuous flow of Tyrode solution was switched to the hypoosmotic solution. For pipette stimulation pipettes were made in the same manner as for patch-clamping RBCs (Thomas et al., 2001), in detail glas pipettes were pulled from glas capillaries (GB150-8P, Science Products, Berlin, Germany) on a DMZ Universal Puller (Seitz, Munich, Germany). Pipettes were prefilled with Tyrode solution and to avoid capillary effects the back end of the pipette was closed with a putty plug. Pipettes were fixed in a patch-clamp pipette holder (HEKA, Lambrecht, Germany). For navigating the pipette a hydraulic micromanipulator WR-6 (Narishige, Tokyo, Japan) was utilized.

### Microfluidic chips

The channels used for the experiments of cells flowing through narrow constrictions are similar to those used in a previous study (Thiam et al., 2016). The custom-made mold used in the study, bearing many combinations of channels and constriction sizes (length and width), was replicated with epoxy resist (Soloplast R123) (Heuzé et al., 2011). PDMS RTV 615 (Momentive) and its curing agent were mixed to a ratio 10:1 (w/w), cast in the mold and cured for 2 h at 70°C. The hardened PDMS was then cut and drilled in the inlets with an 18 G needle and was then bound, after plasma activation (Harrick PDC, 30 s treatment), to a glass-bottom dish (FD35, World Precision Instrument). From the available sizes, the set of channels used in this study were 5 μm high and 8 μm wide, with constrictions of 3 μm wide and 10 μm long. Cells were pushed through the channel by a programmable syringe pump (NE-1000, New Era Pump Systems, Farmingdale, NY, USA) such that RBCs reached a flow speed in the range of 3–5 μm/s.

### Preparation of dorsal skinfold chamber and *in vivo* imaging

#### Animals

The *in vivo* experiments were performed in 12- to 14-week old male C57BL/6 mice with a body weight of 24–26 g. The animals were bred and housed in open cages in the conventional animal husbandry of the Institute for Clinical & Experimental Surgery (Saarland University, Germany) in a temperature-controlled environment under a 12 h/12 h light-dark cycle and had free access to drinking water and standard pellet food (Altromin, Lage, Germany). All experiments were approved by the local governmental animal care committee (approval Number 06/2015) and were conducted in accordance with the German legislation on protection of animals and the NIH Guidelines for the Care and Use of Laboratory Animals (Institute of Laboratory Animal Resources, National Research Council, Washington, USA).

#### Dorsal skinfold chamber model

Red blood cell passage of small capillaries was analyzed in the dorsal skinfold chamber model, which provides continuous microscopic access to the microcirculation of the striated skin muscle and the underlying subcutaneous tissue (Laschke and Menger, 2016). For the implantation of the chamber, the mice were anesthetized by i.p. injection of ketamine (75 mg/kg body weight; Ursotamin®; Serumwerke Bernburg, Bernburg, Germany) and xylazine (15 mg/kg body weight; Rompun®; Bayer, Leverkusen, Germany). Subsequently, two symmetrical titanium frames (Irola Industriekomponenten GmbH & Co. KG, Schonach, Germany) were implanted on the extended dorsal skinfold of the animals in a stepwise procedure, as described previously in detail (Laschke et al., 2011). Within the area of the observation window, one layer of skin was completely removed in a circular area of ~15 mm in diameter. The remaining layers (striated skin muscle, subcutaneous tissue and skin) were finally covered with a removable cover glass. To exclude alterations of the microcirculation due to the surgical intervention, the mice were allowed to recover for 48 h.

#### *In vivo* microscopy

*In vivo* microscopic analyses were performed as previously described (Brust et al., 2014). In detail, the mice were anesthetized and a fine polyethylene catheter (PE10, 0.28 mm internal diameter) was inserted into the A. carotis for application of labeled RBCs. Then, the animals were put in lateral decubital position on a plexiglas pad and the dorsal skinfold chamber was attached to the microscopic stage of an upright microscope (E600; Nikon, Tokyo, Japan) equipped with a 40x, NA 0.8, water immersion objective and a halogen lamp attached to a fluorescein isothiocyanate (FITC) filterset (excitation 465–495 nm, emission 515–555 nm). For labeling RBCs blood samples were taken from siblings and *ex vivo* stained with Fluo-4, AM as described above. Up to 0.5 mL of stained RBCs were transfused directly prior to the imaging experiments. The microscopic images were recorded using a charge-coupled device video camera (iXon Ultra; Andor, Belfast, UK) connected to a PC system at an acquisition speed of 212 images per second (4.5 ms exposure time, 2 × 2 binning, shift speed 0.3 μs, readout rate 17 MHz). For image processing, the black and white pictures were changed into a “fire” look-up table for better visualization.

### Measuring fiterability

Blood samples were centrifuged at 1,000 × g for 20 min. Plasma was aspirated and mixed with phosphate buffered saline (PBS) (1:10). Erythrocytes were mixed with Tyrode solution (1:1) and washed three times (1,000 × g, 5 min). Filterability was tested by a modified method originally developed for the depletion of leukocytes (Beutler et al., 1976; Minetti et al., 2013). Filter paper (Whatman No. 4, GE Healthcare, UK) was pressed in a 3 mL syringe (Omnifix Solo Lure, Braun, Germany) and 200 mg Sigma- and 100 mg Alpha-Cellulose was added. The syringe was filled further with 2 mL Tyrode solution and shaked to allow the cellulose to mix. After the Tyrode drained, the syringe was primed with 2 mL of the diluted plasma. Subsequently, 500 μL of the RBC/Tyrode mixture was added at stopped flow conditions. Another 2 mL of Tyrode solution was carefully added. The flow through the syringe was started and the filtrate collected for exactly 1 min. The amount of RBCs was related to the amount of hemoglobin, which was determined photometrically. To standardize the measurements different forms of hemoglobin were converted to hemiglobincyanid as previously described (Meyer-Wilmes and Remmer, 1956). Absorption was measured at 546 nm (Lambda Bio+, Perkin Elmer, Waltham, MA, USA).

### Image analysis

All image analysis was performed in ImageJ (Wayne Rasband, National Institute of Health, USA). For the images displayed in the figures a look-up table named “fire” was applied. To extract data from the images raw data were used. First a background subtraction was performed (subtraction of an image just without the cells). Then regions of interest (ROI) were defined for each cell or for each position of the cell when analyzing cells in flow. To create graphs of cellular responses we presented the fluorescence intensity as self ratios F/F_o_, i.e., the entire fluorescence trace was divided by the fluorescence value at the beginning of the recording to normalize the starting conditions and to compensate for cellular differences in dye loading or hemoglobin concentration (Kaestner et al., 2006).

### Statistics

For all statistical analysis the Gaussian distribution of the dataset was checked by the D'Agostino and Pearson omnibus normality test. For data with Gaussian distribution the mean value ± the standard error of mean (SEM) was plotted as column graphs. Testing for significant differences was performed with a paired *t*-test, wheras *p* < 0.0001 was denoted with four stars (^****^) and non-significant differences abbreviated with “ns.”

Datasets with a non-Gaussian distribution were visualized as box-plots representing the median and the 25–75th percentile and whiskers representing the 5–95th percentile. Significance was tested with Mann-Whitney test and significance levels were indicated with three stars (^***^) for *p* < 0.001, with one star (^*^) for *p* < 0.05 and “ns” for not significant.

All graph presentations and statistical tests were performed in GraphPad Prism (GraphPad Software, La Jolla, CA, USA).

## Results

### RBC Ca^2+^-response after mechanical stimulation in stasis

**Figure 1:**
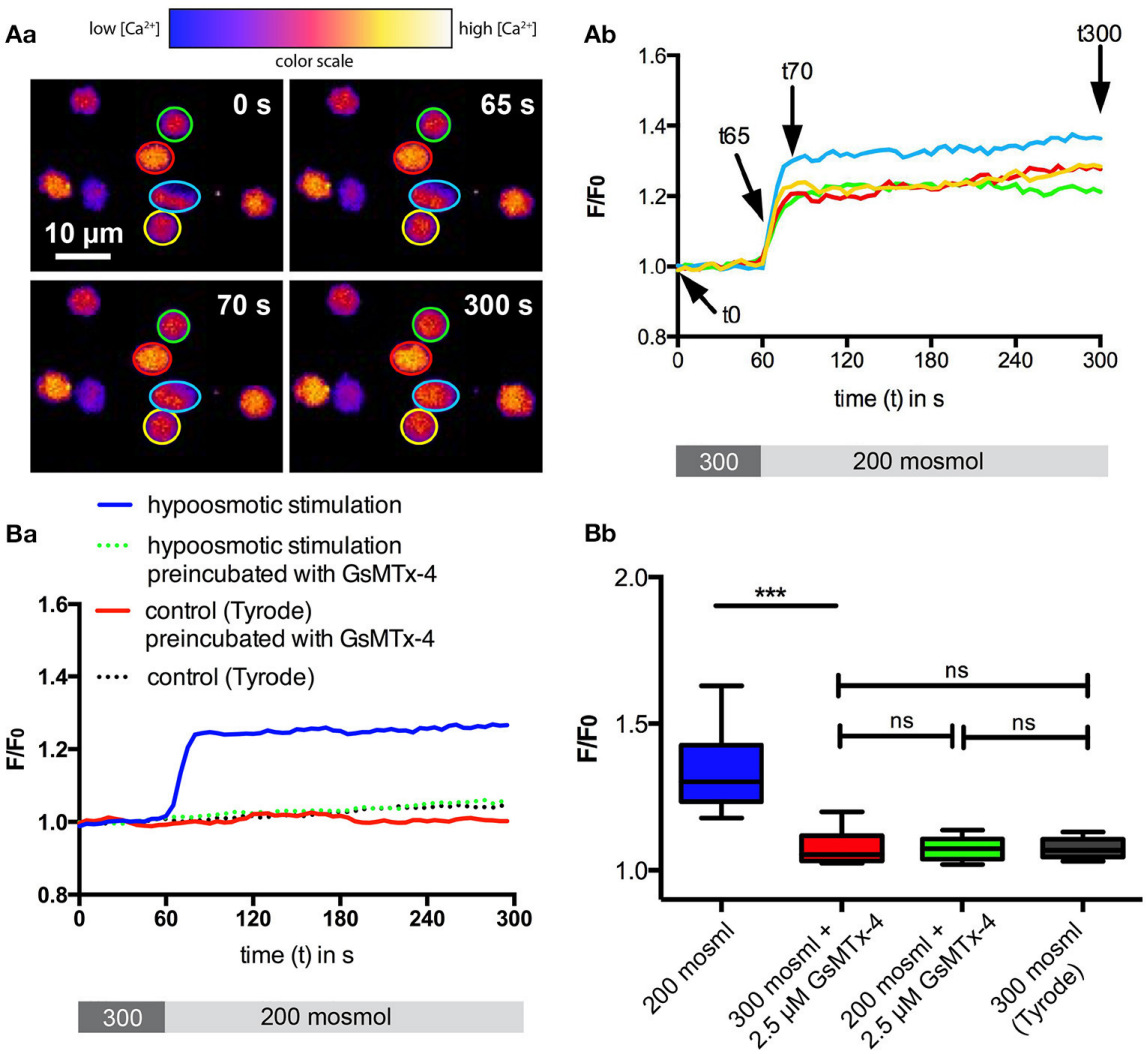
Ca^2+^-signaling of RBCs after hypoosmotic treatment. RBCs in Tyrode solution were exposed to 200 mosmol solution. **(A)** depicts confocal example images at four time points **(Aa)** and example traces **(Ab)** indicating the time points displayed in **(Aa)** with arrows. The cellular Ca^2+^-response as fluorescence intensity (F/F_0_) of the Ca^2+^-fluorophor Fluo-4 is plotted over time. The color-coded regions of interest in **(Aa)** correspond to the colors of the traces in **(Ab)**. Below the graph in **(Ab)** the protocol of the solution application (60 s, 300 mosmol followed by 240 s 200 mosml) is provided. **(B)** shows the statistical analysis. For each condition between 160 and 210 cells from three different donors were analyzed. The median fluorescence trace (F/F_0_) over time of all cells is depicted in **(Ba)**. Below the graph the protocol of the solution application (60 s, 300 mosmol followed by 240 s 200 mosml) is provided. The analysis of the cellular maximal response (F/F_0_) is depicted in **(Bb)**. The response under hypoosmotic conditions in the absence of GsMTx-4 is significantly different relative to all other conditions measured (*p* < 0.001; ^***^).

In an initial experiment we aimed to check if basic mechanical stimulation by osmotic swelling leads to an activity of mechano-sensitive channels in RBCs. We challenged the cells with a 200 mosmol solution and used the change in intracellular free Ca^2+^ as a read-out parameter. The results of the microscopic measurements using the Ca^2+^-fluorophore Fluo-4 are summarized in Figure 1. In contrast to hormonal-like stimulations (Wang et al., 2013), RBCs showed qualitatively a very homogeneous response, i.e., all cells responded immediately with an increase in intracellular Ca^2+^, just the extent of the increase varied between the cells. This Ca^2+^-increase could be completely blocked by preincubation of the RBCs with 2.5 μM GsMTx-4 (Figure 1Bb, green box), a widely used inhibitor of Piezo1 (Bae et al., 2011), whereas GsMTx-4 itself had no effect on cells in isosmotic solution (Figure 1Bb, red box).

**Figure 2:**
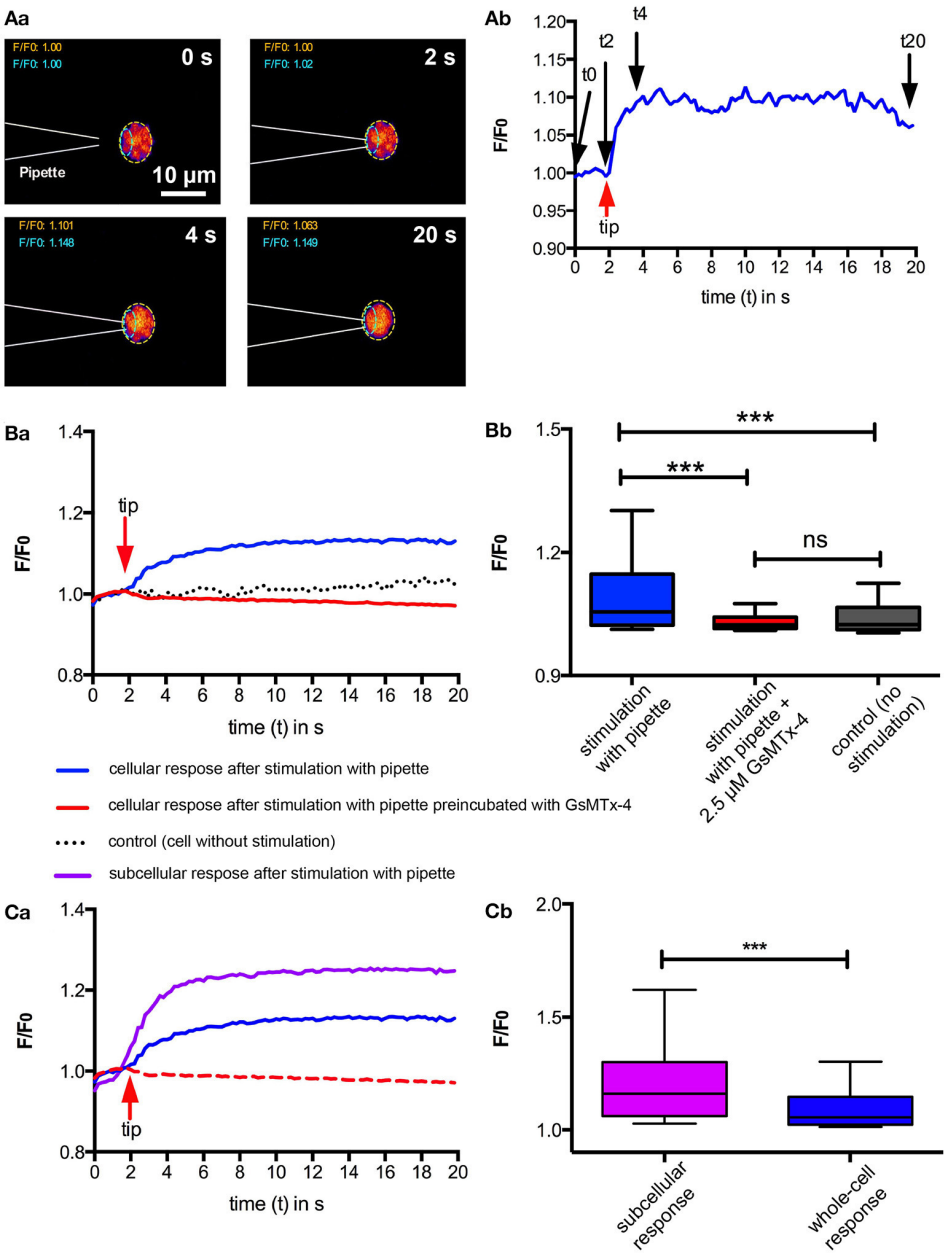
Ca^2+^-signaling of RBC after local mechanical stimulation. Individual RBCs were mechanically stimulated using a micropipette. **(A)** exemplifies a typical experiment. Fluorescent confocal images of a Fluo-4 loaded cell when touched by a micropipette at four time points are shown in **(Aa)**. The color scale is the same as in Figure 1Aa. Since the pipette is not visible in the fluorescent image, its position is drawn in the images by dashed white lines. The fluorescence intensity (F/F_0_) of the region of interest (ROI) indicated by the dashed yellow line in **(Aa)** is plotted in **(Ab**) indicating the time point when the pipette touches the cell (red arrow) and the time points of the images displayed in **(Aa)** (black arrows). **(B)** depicts the statistical analysis comparing pipette stimulation in the absence and presence of GsMTx-4 with unstimulated RBCs. For each condition between 74 and 76 cells from three different donors were analyzed. The median fluorescence trace (F/F_0_) over time of all cells is depicted in **(Ba)**. The red arrow indicates the time point when the pipette was touching the RBC. The analysis of the cellular maximal response (F/F_0_) is depicted in **(Bb)**. The response of the mechanical stimulation in the absence of GsMTx-4 is significantly different relative to all other conditions measured (*p* < 0.001; ^***^). **(C)** Compares the cellular response of 76 RBCs as depicted in **(B)** with the local (subcellular) fluorescence intensity at the spot where the micropipette is touching the cell as indicated by the ROI outlined with cyan dashed lines in **(Aa)**. The median fluorescence trace (F/F_0_) over time of all cells is depicted in **(Ca)**. The red arrow indicates the time point when the pipette was touching the RBC. For a better comparison the blue and the red lines are replotted from **(Ba)**. The comparison of the local (subcellular) and cellular (whole-cell) maximum response (F/F_0_) is depicted in **(Cb)**. The local response is significantly higher than the whole-cell response (*p* < 0.001; ^***^).

Next we tested if local mechanical stimulation by poking an individual RBC with a micropipette would also lead to an increase in intracellular Ca^2+^. The results of these experiments are presented in Figure 2. Touching the cell with the micropipette resulted in an immediate increase in intracellular Ca^2+^, which could be inhibited by preincubation with 2.5 μM GsMTx-4 (Figures 2A,B). Furthermore, we could even identify the spot where the micropipette was touching the cell as the source of the Ca^2+^-entry, because maximal intensity values in the vicinity of this spot (Figure 2Aa, cyan region of interest) reached higher than the values considering the full cellular confocal cross section (Figure 2C). To exclude the increase in fluorescence intensity was caused by an artifact (e.g., cellular compression and putative increase in Fluo-4 concentration) we performed control experiments with a simultaneous Calcein Red-Orange staining, a dye that is insensitive to Ca^2+^ and other ions but still has a cytoplasmic localisation. The Calcein Red-Orange fluorescence was constant during the entire measurement indicating (i) that the cell membrane was not damaged and (ii) lack of volume related artifacts in the fluorescence intensity.

### RBC Ca^2+^-response while passing through constrictions in microfluidic channels

**Figure 3:**
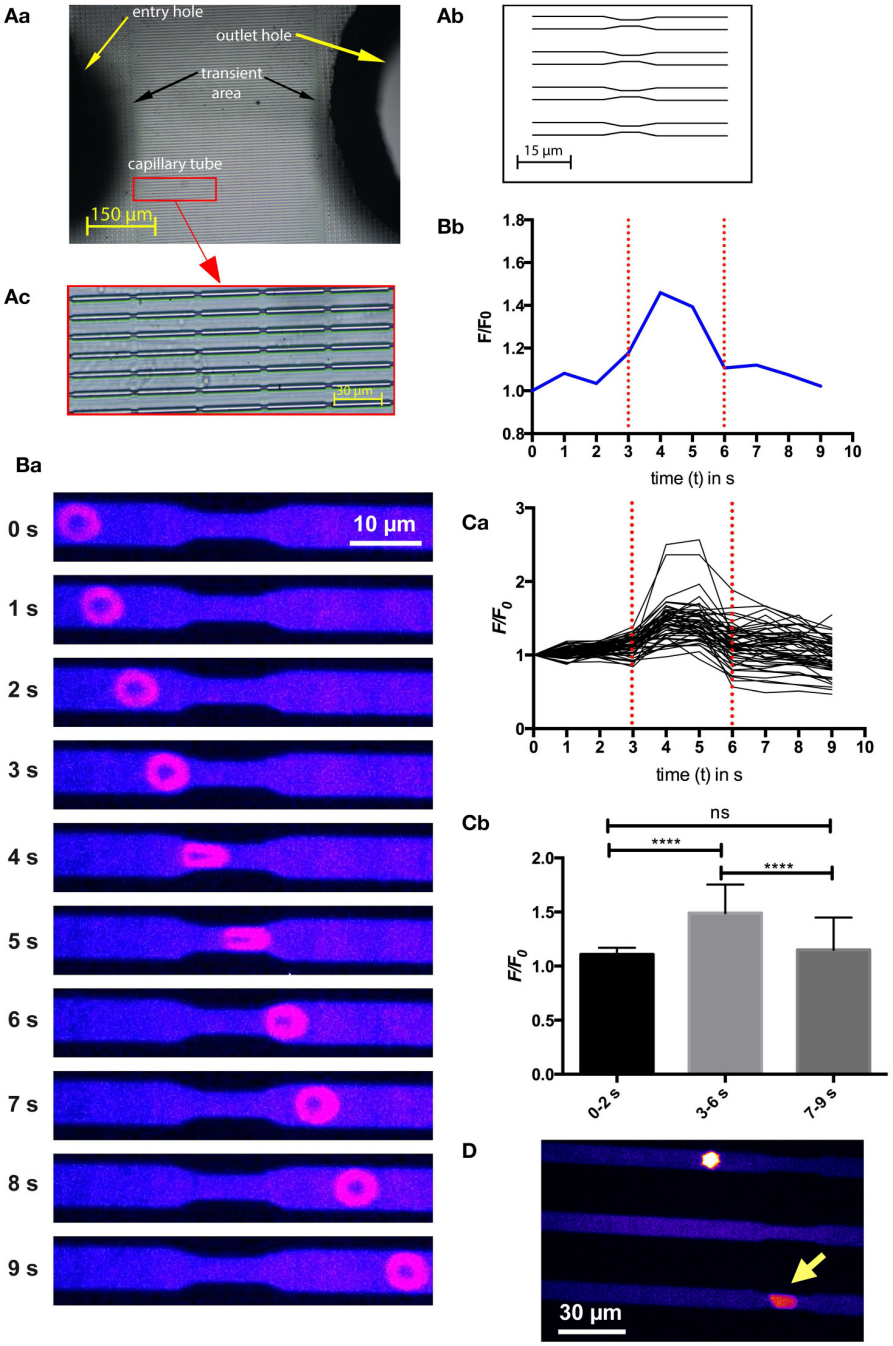
Ca^2+^-signaling of a RBC passing through constrictions in a microfluidic chip. **(A)** depicts the design of the microfluidic chip used. A photographic overview with labeled parts of the chip is given in **(Aa)**, whereas a schematic overview of parallel channels with constrictions to simulate capillaries is drawn in **(Ab)**. In contrast, the real appearance of the channels with repetitive constrictions is displayed in **(Ac)**. **(B)** exemplifies a typical experiment. Fluorescent confocal images of a Fluo-4 loaded cell when passing through the microfluidic channel at 10 time points are shown in **(Ba)**. The color scale is the same as in Figure 1Aa. The fluorescence intensity (F/F_0_) of the RBC at the 10 time points shown in **(Ba)** is plotted in **(Bb)** indicating the start and the end of the constriction by red dotted lines. A video of this experiment is available in the supplemental material (Supplemental Video 1). **(C)** depicts the analysis of 52 cells measured while passing through the constriction. The fluorescence intensity (F/F_0_) traces of all measured RBCs are plotted in **(Ca)**. The statistical analysis of the maximal fluorescence intensity (F/F_0_) at time points before the constriction (0–2 s), when passing through the constriction (3–6 s) and after passing through the constriction (7–9 s) is depicted in **(Cb)**. The increase in Ca^2+^, while passing through the constriction is highly significant (*p* < 0.0001; ^****^). **(D)** Shows an image of RBCs treated with 1 μM GsMTx-4 getting stuck in the microfluidic channels. The yellow arrow points to a cell sticking in a constriction. To better judge the resting state of the cell please refer to Supplemental Video 2.

Since RBCs experience a mechanical stimulation when small capillaries or interendothelial slits in the spleen, we designed a microfluidic chip that would mimic such a constriction (Figure 3A). The measurement procedure as well as the statistical analysis are depicted in Figures 3B,C as well as in Supplemental Video 1. The cells indeed showed a transient increase in intracellular Ca^2+^ when passing through the constriction. In similarity to the previous experiments (Figures 1, 2) all cells reacted with such a Ca^2+^ increase just to a different degree. The hypothesis that we are facing a mechanism of adaptive volume regulation initiated by mechano-sensitive channels is supported by attempts to perform the same experiments as presented in Figure 3B with RBCs preincubated with GsMTx-4. Under these conditions the channels of the microfluidic chip were clogged immediately preventing any cellular analysis (Figure 3D and Supplemental Video 2). This was confirmed for GsMTx-4 concentrations in the range of 2.5 – 0.1 μM.

### *In vivo* RBC Ca^2+^-response while passing through capillaries

**Figure 4:**
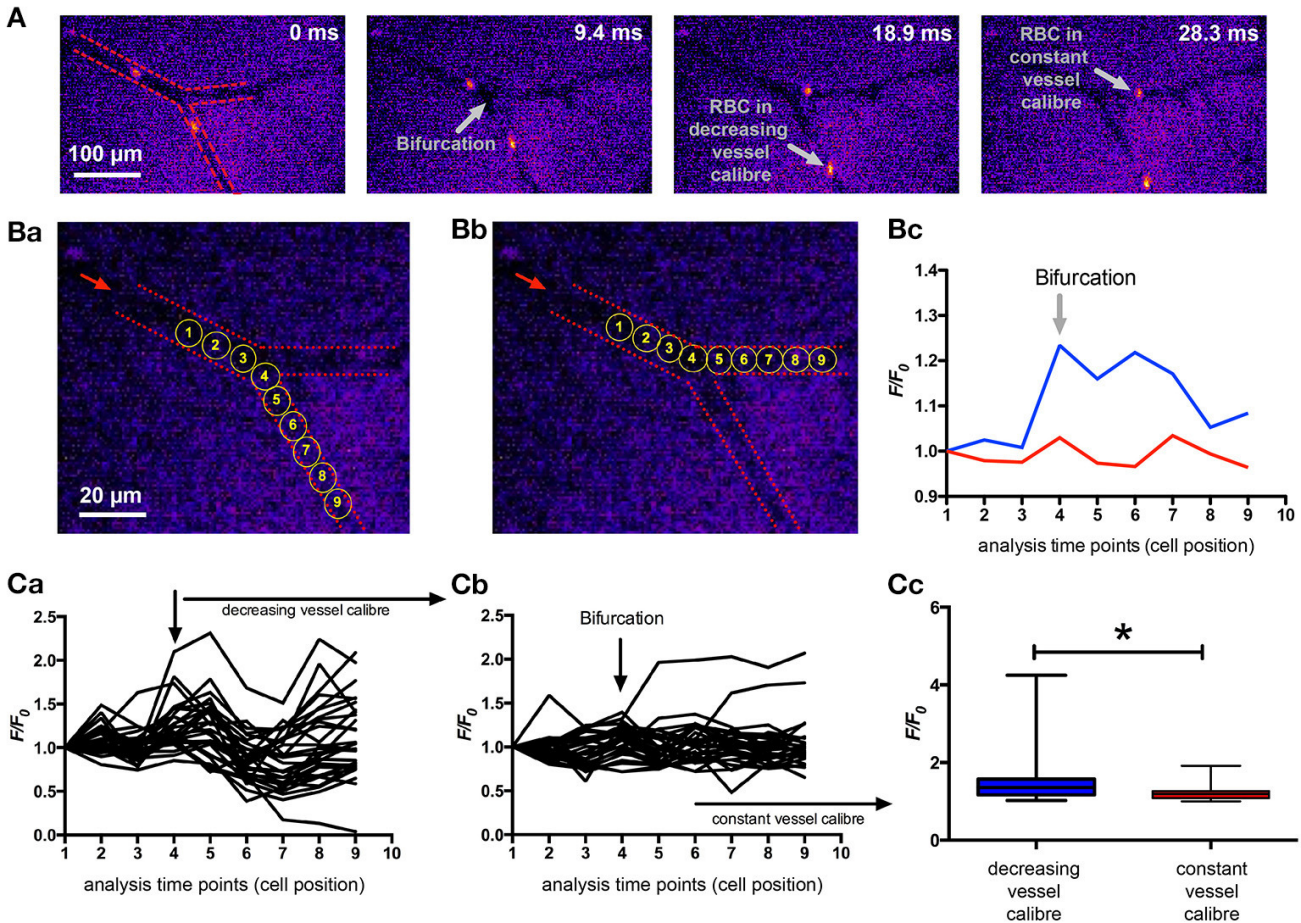
*In vivo* Ca^2+^-signaling of mouse RBCs when passing through capillaries. Mouse RBCs were *ex vivo* stained with Fluo-4 and then re-injected into the mouse circulation. Fluorescence imaging of capillaries was performed in the dorsal skinfold chamber. **(A)** Shows representative snapshots of RBCs passing a bifurcation. For the 30 ms sequence only every second recorded image is presented. For a better orientation the vessel walls are indicated by red dashed lines in the leftmost image and further annotations (gray) are added in the other images. **(B)** depicts the positions where fluorescence intensity (F/F_0_) was analyzed for a decreasing vessel caliber **(Ba)** and for a constant vessel caliber **(Bb)** of the same example section as in **(A)**. The dashed red lines mark the vessel walls, the red arrow indicates the blood flow direction and the yellow circles depict the analysis positions which are plotted in the following diagrams. Example fluorescence traces of the two cells analyzed as pointed out in **(Ba,Bb)** are shown in **(Bc)**. Videos of these two example cells are available in the supplemental material (Supplemental Videos 3, 4, respectively). **(C)** depicts the analysis of 30 cells passing through a capillary with decreasing vessel caliber and 28 cells passing through a capillary with constant vessel caliber. Analysis was performed at three vessel-bifurcations in two mice. The fluorescence intensity (F/F_0_) traces of all measured RBCs passing through a capillary with decreasing vessel caliber is plotted in **(Ca)**, while the traces of all measured RBCs passing a capillary with constant vessel caliber is plotted in **(Cb)**. The statistical analysis of the maximal fluorescence intensity (F/F_0_) of RBCs from both groups is depicted in **(Cc)**. The increase in Ca^2+^, while passing through a vessel with decreasing caliber is significant (*p* = 0.014; ^*^).

To answer the question if the results from the microfluidic channels are of physiological relevance, we aimed for *in vivo* measurements and chose the mouse model utilizing a dorsal skinfold chamber for the optical imaging. Mouse RBCs were stained with Fluo-4 *ex vivo* and then reinjected into the circulation. The fast movement of the RBCs in the capillaries required high speed fluorescence imaging at a frame rate exceeding the 200 Hz. We chose imaging positions of capillary bifurcations, where one vessel showed a decreasing caliber while the other one remained fairly constant in order to have constrictions and a control condition in the same image sequence. Representative recordings are depicted in Supplemental Videos 3 and 4. Figure 4 summarizes the analysis of RBCs in three of such bifurcations (from 2 mice). We could identify a significantly higher Ca^2+^ in the RBCs passing through the vessel with the decreased caliber compared to control conditions (Figure 4C).

### Measurements of RBCs filterability

**Figure 5:**
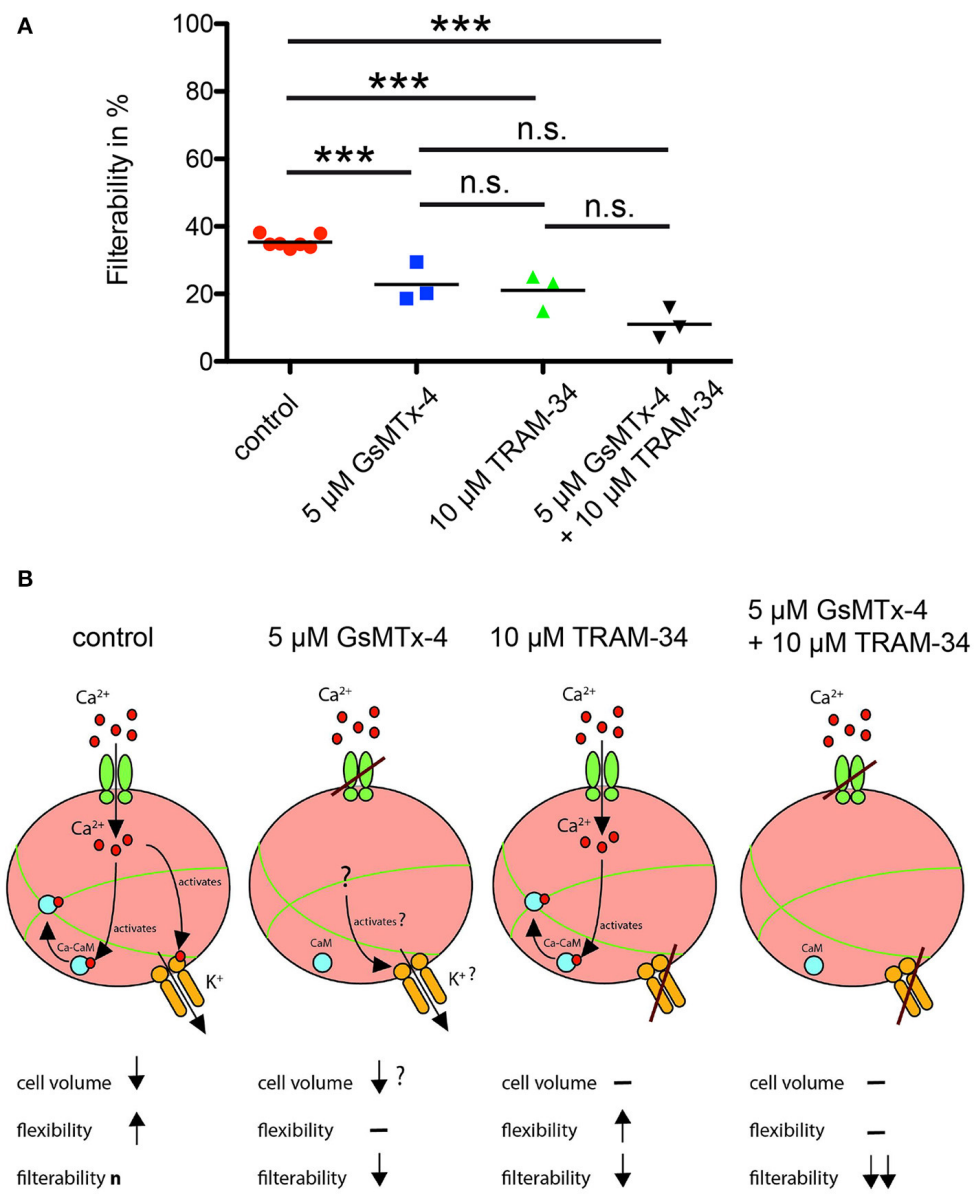
RBC filterability and putative mechanisms. **(A)** shows measurements of the filterability of RBCs under control conditions (red circles) and in the presence of GsMTx-4, a blocker of the Piezo1 ion channel (blue squares), in the presence of TRAM-34, an inhibitor of the Gardos-channel (green triangles) and in the presence of both drugs (black triangles; ^***^p < 0.001). A summary of these data was already published in a different context (Fermo et al., 2017). **(B)** visualizes the putative mechanism for all four experimental conditions depicted in **(A)**. Under control conditions interaction of the RBC with the cellulose activates mechanosensitive channels such as Piezo1 (green channel symbol), Ca^2+^ (red circles) can enter the cell. Ca^2+^ activates the Gardos-channel (orange channel symbol) and by formation of the calcium-calmodulin complex (Ca-CaM; associated blue and red circles) loosens the cross-linked spectrin tetramers (green lines). For a more detailed description of this process see Discussion. These processes will reduce the cell volume and increase the flexibility of the cell resulting in the “normal” filterability n. If GsMTx-4 blocks the Piezo1, the Ca^2+^-entry pathway is impaired and the above-described process is diminished. However, it is not clear if Ca^2+^-permeable channels other than Piezo1 are involved, and hence a slight adaptation in cell volume occurs. If TRAM-34 blocks the Gardos-channel, Ca^2+^ may still enter the cell through Piezo1, which should still allow the modification of the spectrin network but not the volume adaptation resulting in decreased filterability. If both Piezo1 and Gardos-channel are inhibited all mechanisms described above are blocked with the expected consequence for the RBCs' filterability.

In order to test if our microscopic results have a macroscopic implication, we measured the filterability of human blood samples and compared it with samples preincubated with 5 μM GsMTx-4 or 10 μM TRAM-34, a Gardos-channel inhibitor, or with both substances simultaneously (Figure 5A). Any of the applied pharmacological interventions, i.e., any impairment of the proposed mechano-sensitive volume regulation (Figure 5B), significantly reduced the RBC filterability.

## Discussion

**Figure 6:**
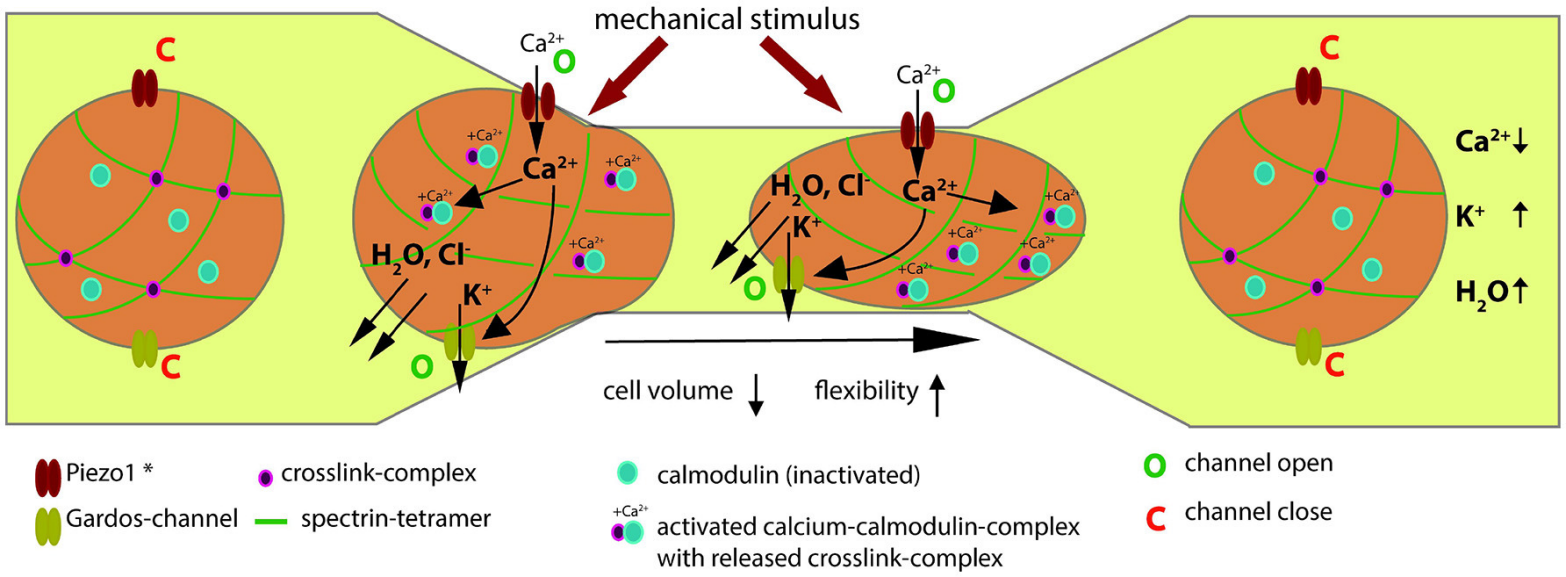
Evidence based hypothesis of the Ca^2+^-signaling mechanism when RBCs pass capillaries. The description of a RBC passing through a constriction is described from left to right. Under laminar flow of RBCs in the vessel, for both Piezo1 and the Gardos-channel the open probability is very low and they can be regarded as being in a closed state (denoted by a “C” next to the channel symbols). ^*^In this scheme Piezo1 denotes the most probably involved molecular player but could also be a different mechanosensitive mechanism. As the RBC enters a constriction and the cell comes in mechanical contact with the vessel wall, this mechanical stimulus activates Piezo1 (denoted by a “O” next to the channel symbol). Since Piezo1 is a non-selective cation-channel (Gottlieb and Sachs, 2012) and in view of the tremendous gradient for Ca^2+^ across the membrane (Tiffert et al., 2003), Ca^2+^ will enter the cell. This will activate the Gardos-channel (denoted by a “O” next to the channel symbol) and the calcium-calmodulin complex (Ca-CaM) will be formed (associated blue and pink circles). Activation of the Gardos-channel will result in loss of K^+^, Cl^−^ and water, i.e., in cell shrinkage. The Ca-CaM destabilizes the actin—addusin—Band 4.1 complex and the cross-linked spectrin network becomes more flexible (interrupted green lines). For a more detailed description of this process see Discussion. Both processes facilitate the passage of the RBC trough the constriction. Both, Piezo1 and Gardos-channels are likely to inactivate and close (denoted by a “C” next to the channel symbols). Na^+^/K^+^-pump and Ca^2+^-pump (not shown in scheme) will restore the original ion concentrations and such the RBC volume will return to its original size. Likewise the Ca-CaM will inactivate resulting in the dissociation of the calmodolin (blue circles) and thus the cross-linking of the spectrin tetramers (pink circles at cross-section of green lines).

We investigated RBCs after various modes of mechanical stimulation: osmotic swelling (Figure 1), poking individual cells with a micropipette (Figure 2) and squeezing cells through a constriction in a microfluidic channel (Figure 3). To transfer this scenario to *in vivo* conditions we used a mouse model (Figure 4) and finally we performed macroscopic measurements on cell populations (Figure 5). All these measurements gave consistent data supporting our hypothesis outlined in Figure 6. When RBCs are passing through a capillary with a diameter smaller than the cross-section of a RBC or interendothelial slits in the spleen, they need to be super-deformable and undergo a transient volume decrease. We could show that all kinds of mechanical stimulation resulted in an intracellular increase in Ca^2+^. We did not demonstrate the molecular identity mediating the Ca^2+^-entry but based on the knowledge of mechano-sensitive channels in the human RBC (Cinar et al., 2015; Kaestner, 2015), the sensitivity of our measurements for GsMTx-4 (Bae et al., 2011 and Figures 1B, 2B, 5) and the comparison of our results with knock-out animals (Faucherre et al., 2014; Cahalan et al., 2015) it is very likely that Piezo1 is the mechano-sensitive “Ca^2+^-source.” However, involvement of other mechano-sensitive transport cannot be excluded. NMDA-receptors which are also present in the RBC-population (Makhro et al., 2013) show a mechano-sensitivity but it is not sensitive to GsMTx-4 (Maneshi et al., 2017).

Considering the long lasting Ca^2+^-signals in Figures 1, 2, we have to admit that in both cases the mechanical stimulation (osmotic pressure and pipette touching the cell) lasted during the entire experiment, Piezo1 activation would suggest a transient Ca^2+^-entry. If Ca^2+^ entry would have been carried exclusively through the Piezo1 channel, due to the inactivation kinetics of the channel, such an entry would suddenly stop. Indeed the Ca^2+^-level remains constant after the initial increase pointing to an equilibrium between Ca^2+^-extrusion by the Ca^2+^-pump and Ca^2+^ entry. A special case for such an equilibrium could be Piezo1 inactivation and the failure of the Ca^2+^-pump, e.g., by Ca^2+^-mediated destruction of the pump fuelling ATP-pools (Chu et al., 2012). For a transient mechanical stimulation, e.g., when passing through a constriction, (Figures 3, 4) the associated Ca^2+^ signal as expected was also transient.

Once Ca^2+^ enters the RBCs it initiates a plethora of processes in a concentration dependent manner (Bogdanova et al., 2013). One of them (responsible for the dehydration—compare Introduction) is the Gardos-channel, which was also challenged by application of TRAM-34 in the measurements of the filterability (Figure 5). In addition to the Gardos-channel mediated dehydration, in Figure 6 we render a further Ca^2+^-dependent process, the Ca^2+^ binding to calmodulin, forming the Ca^2+^-calmodulin complex (Ca-CaM). Protein Band 4.1 and adducin interact with Ca-CaM. Adducin binds to actin blocking elongation of the fast-growing (barbing) ends of actin filaments within the junctional complexes. Interaction with Ca-CaM down-regulates capping activity of adducin regulating thereby actin filament assembly (Kuhlman et al., 1996). Furthermore, adducin tetramers participate in docking of carbonic anhydrase II to band 3 tetramers. When interacting with the band 3 dimers anchoring the spectrin network to the membrane, the junctional complex becomes a part of bigger multi-protein complexes known as 4.1R-complexes. Interaction of the 4.1R-complex with Ca-CaM triggers the reduction of the affinity of this protein to all interacting partners. As a result, spectrin network interaction with the integral proteins becomes loose and finally the RBC show an increased flexibility (Bogdanova et al., 2013).

Our data allow a transfer of knowledge originally achieved with knock-out approaches of Piezo1 in zebrafish (Faucherre et al., 2014) and mice (Cahalan et al., 2015) to human RBCs as outlined above. Furthermore, comparing our micropipette poking experiments with very similar patch-clamp experiments with human RBCs (Dyrda et al., 2010), showing a mechanical stimulation followed by the activation of the Gardos-channel is in support of the Piezo1—Gardos-channel interplay for transient volume adaptation.

## Author contributions

LK defined the study, JD, FL, ML, and LK planned the experiments. JD, ET, LH, PP-K, ML, and LK performed the acquisition and analysis. JD, PP-K, and LK interpreted the data. JD drafted the figures and LK drafted the manuscript. ET, LH, PP-K, FL, and ML critically revised the manuscript. All authors approved the final version of the manuscript.

### Conflict of interest statement

The authors declare that the research was conducted in the absence of any commercial or financial relationships that could be construed as a potential conflict of interest.
