# Supplementary material for: Red Blood Cell Passage of Small Capillaries Is Associated with Transient Ca2+-mediated Adaptations
Source: Front Physiol. 2017 Dec 5;8:979. doi: 10.3389/fphys.2017.00979 (PMC5723316; doi:10.3389/fphys.2017.00979)
Supplement: Supplementary file 5 [file DataSheet1.PDF]

Supplementary Material

Red blood cell passage of small capillaries is associated with transient Ca^{2+}-mediated adaptations

Jens G. Danielczok, Emmanuel Terriac, Laura Hertz, Polina Petkova-Kirova, Franziska Lautenschläger, Matthias W. Laschke, Lars Kaestner*

*** Correspondence:** Lars Kaestner: lars_kaestner@me.com

1 Supplementary Videos

Supplemental Video 1

This video shows a confocal recording of a RBC passing a microfluidic constriction similar as the cell depicted in Figure 3Ba. The cell is followed by a 2nd smaller cell, which due to its size experiences less of a mechanical stimulation and hence depicts a minor Ca^{2+} signal.

Supplemental Video 2

This video shows in extension of Figure 3D a confocal recording of RBCs treated with 1 µM GsMTx-4 that got stuck in the microfluidic channels.

Supplemental Video 3

This video shows a high speed imaging sequence (212 images/s) of a RBC (as analysed in Figure 4Bc) passing a bifurcation entering a vessel with decreasing calibre. For orientation compare Figure 4Ba.

Supplemental Video 4

This video shows a high speed imaging sequence (212 images/s) of a RBC (as analysed in Figure 4Bc) passing a bifurcation entering a vessel with constant calibre. For orientation compare Figure 4Bb.
